# Finite Element Simulation of Acoustic Emissions from Different Failure Mechanisms in Composite Materials

**DOI:** 10.3390/ma17246085

**Published:** 2024-12-12

**Authors:** Manoj Rijal, David Amoateng-Mensah, Mannur J. Sundaresan

**Affiliations:** Department of Mechanical Engineering, North Carolina A & T State University,1601 E. Market Street, Greensboro, NC 27411, USA; damoatengmensah@aggies.ncat.edu (D.A.-M.); mannur@ncat.edu (M.J.S.)

**Keywords:** acoustic emission (AE), damage mechanisms in composites, cross-ply laminate, finite element analysis (FEA)

## Abstract

Damage in composite laminates evolves through complex interactions of different failure modes, influenced by load type, environment, and initial damage, such as from transverse impact. This paper investigates damage growth in cross-ply polymeric matrix laminates under tensile load, focusing on three primary failure modes: transverse matrix cracks, delaminations, and fiber breaks in the primary loadbearing 0-degree laminae. Acoustic emission (AE) techniques can monitor and quantify damage in real time, provided the signals from these failure modes can be distinguished. However, directly observing crack growth and related AE signals is challenging, making numerical simulations a useful alternative. AE signals generated by the three failure modes were simulated using modified step impulses of appropriate durations based on incremental crack growth. Linear elastic finite element analysis (FEA) was applied to model the AE signal propagating as Lamb waves. Experimental attenuation data were used to modify the simulated AE waveforms by designing arbitrary magnitude response filters. The propagating waves can be detected as surface displacements or surface strains depending upon the type of sensor employed. This paper presents the signals corresponding to surface strains measured by surface-bonded piezoelectric sensors. Fiber break events showed higher-order Lamb wave modes with frequencies over 2 MHz, while matrix cracks primarily exhibited the fundamental S_0_ and A_0_ modes with frequencies ranging up to 650 kHz, with delaminations having a dominant A_0_ mode and frequency content less than 250 kHz. The amplitude and frequency content of signals from these failure modes are seen to change significantly with source–sensor distance, hence requiring an array of dense sensors to acquire the signals effectively. Furthermore, the reasonable correlation between the simulated waveforms and experimental acoustic emission signals obtained during quasi-static tensile test highlights the effectiveness of FEA in accurately modeling these failure modes in composite materials.

## 1. Introduction

Acoustic emission (AE) monitoring can serve as an effective structural health monitoring tool if accurate quantification and the ability to distinguish different types of AE sources are feasible. Distinguishable patterns of the AE waveforms corresponding to various sources, such as fiber breaks, fiber pullout, fiber debonding, matrix crack, and delamination in carbon fiber composites, along with other extraneous noise, must be established to monitor and quantify damage in such material systems. Several studies have been conducted on quantifying sources in carbon fiber-reinforced polymer (CFRP) either by evaluating the features of acquired acoustic emission signals or the whole signal recorded by the AE sensors.

Composite materials exhibit diffuse and complex damage accumulation processes, and the nature of the damage is influenced by the interactions between the composite’s fiber and matrix subsystems, the type of loading applied, and the inherent characteristics of the structure [1]. The main failure modes in composite materials include matrix cracking, delamination, and fiber breaks, while the damage to the fibers in load-carrying directions is considered critical to the ultimate strength of the material in tension. An extensive body of literature exists on the accumulation of different failure modes under static and fatigue loading. Several acoustic emission studies have focused on a single dominant damage mechanism (SDDM), which can be brought upon by a combination of specific loading and ply orientation. For instance, Gutkin et al. [2] investigated failure processes in several configurations of composite specimens and identified acoustic emission patterns corresponding to individual failure modes. In one of their tests of cross-ply tensile coupons, damage initiated as transverse matrix cracks in the 90^0^ layers, which later developed as edge delaminations. In contrast, double cantilever beam (DCB) and four-point end-notched flexure (4-ENF) tests primarily exhibit delamination failure modes. Similarly, Brunner [3] found that cross-ply laminates exhibit transverse matrix cracks as the initial SDDM during early loading stages, which eventually saturate and lead to delaminations. In multidirectional laminates such as [0/45_2_/90]_s_, under quasi-static loading, Yokozeki et al. [4] also found that the initial transverse matrix cracks in 90^0^ laminae initiate gradually increasing levels of damage in other off-axis plies. Takeda and Ogihara [5] investigated the initiation of delaminations from the tips of transverse matrix cracks in toughened cross-ply CFRP laminates and the resulting loss in laminate stiffness. Jamison [6] detailed various damage modes, including transverse matrix cracks, axial matrix splits, delaminations, and fiber breaks under fatigue and monotonic tensile loading in unidirectional and cross-ply laminates. He noted that specimens subjected to monotonic tensile loading experienced lower damage density than those subjected to fatigue loading. He also observed fiber breaks in 0^0^ plies adjacent to the matrix crack tips in 90^0^ plies. Beaumont et al. [7] provide a detailed discussion of damage in composite laminates supported by direct observation. They describe how the individual failure modes interact, including the transition from matrix cracks to delamination and fiber breaks, and how they are influenced by the local stress state at the crack tip. They noted that the combination of in-plane shear stress and normal stress in the vicinity of the matrix crack causes delamination evolution from transverse matrix cracks.

Kumar et al. [8] analyzed the progression of fiber breaks in unidirectional aerospace-grade composites under tension using fractographic images, revealing unstable fractures caused by the failure of multiple adjacent fibers. They also noted that strong interfacial bonding between fiber and matrix could facilitate the propagation of cracks from fiber breaks into the matrix. Scott et al. [9] used computed tomography to study the progressive damage under monotonic load in a notched cross-ply specimen. They provided detailed information about the rate of increase in density of transverse matrix cracks, fiber breaks, delaminations, and longitudinal splits. They observed that distributed individual fiber breaks and small clusters formed at lower load levels, while larger clusters appeared close to the ultimate load.

While several AE parameters have been extensively used in differentiating the failure modes, this approach has limitations as acquisition parameters and sensor fidelity highly influence the calculated AE features and may not reliably indicate the underlying failure events associated with individual waveforms [10]. Surgeon and Wevers [11] provide an efficient classification method based on the proportion of individual Lamb wave modes, such as extensional (symmetric) and flexural (anti-symmetric) modes, present in the acoustic emission waveform generated by different failure mechanisms. Scholey et al. [12] have also used such modal acoustic emission analysis to distinguish failure modes such as matrix crack and delamination in quasi-isotropic laminate, with matrix crack having dominant S_0_ and delamination having dominant A_0_ mode. Additionally, the recorded AE signals are significantly affected by the attenuation related to the damping properties of carbon fiber-reinforced plastics (CFRP) [13,14]. Asamene et al. [15] experimentally determined the frequency and mode-dependent attenuation of Lamb waves along different propagating directions in cross-ply and quasi-isotropic laminates and found that flexural waves undergo significantly more attenuation than extensional modes. Furthermore, Ono and Gallego [16] assessed Lamb wave attenuation across different laminate configurations (unidirectional, cross-ply, and quasi-isotropic), highlighting the pronounced attenuation of the A_0_ mode and the entire waveform, with peak amplitudes diminishing exponentially with increasing distance from the source.

While finite element modeling has proven effective for simulating Lamb wave modes in metals and composites, it has also been applied to model damage in CFRP composites [17]. Many studies have used finite element modeling. However, the FEM models used in these simulations must accommodate ultrasonic waves with higher frequency ranges (MHz). Consequently, various parameters must be carefully defined to ensure accurate outcomes, including the minimum time step, element size, and type. Mesh independence analysis is recognized as a reliable method for determining these parameters. Patil et al. [18] conducted a mesh convergence analysis on an automobile component, proposing that a coefficient of variation below 5% is sufficient to achieve convergence. Additionally, the traditional FEM approach is limited to second-order shape functions (quadratic), which restricts its ability to capture higher modes and, hence requiring finer elements to capture these modes. To address this, higher-order FEM schemes have been developed for simulating the propagation of higher-order modes in guided waves [19]. L. Wang and Yuan [20] effectively utilized FEM to model Lamb wave propagation and generate dispersion curves in quasi-isotropic laminates. Ohtshu [21] found that in numerical simulations of acoustic emission from crack growth, the step function type of impulses generates waveforms that resemble the experimentally observed acoustic emission waveforms. Girão Coelho [22] successfully employed FEM models to simulate Mode I delamination in double cantilever beam (DCB) specimens and Mode II delamination in end notched fixture (ENF) specimens of unidirectional laminates, achieving good agreement with experimental findings. Furthermore, F. Wang et al. [23] investigated the tensile behavior of unidirectional CFRP composites using a 2D FEM model, revealing that the ultimate strength of the composites is influenced by fiber strength statistics and stress distribution resulting from progressive microdamage. Besides FEM, there are other numerical models for modeling Lamb wave-based damage detection in composite laminates [24]. Le et al. [25] used the discrete element method (DEM) for modeling delamination, fiber/matrix debonding, and matrix cracks in composites. Unlike conventional FEM, which uses triangular or quadrilateral elements, DEM uses spherical (3D), circular (2D), or polyhedral shapes that interact by contact, spring and damper links, or by cohesive beams, and hence can serve as very useful numerical tools for modeling the behavior of granular and particulate material. Li et al. [26] used the spectral finite element method (SFE) to analyze Lamb wave propagation in composite laminates, which uses spectral elements with higher-order shape functions, namely, Lagrange polynomials. While modeling complex geometries using SFE was challenging, this approach reduced the computational time compared to the conventional FEA model.

This study uses finite element analysis to model different damage mechanisms in a cross-ply carbon fiber epoxy thermoset with a lay-up sequence of [0/90]_3s_ using different source time functions based on the source duration of these AE events. Similarly, attenuation is incorporated in the FEM-generated waveforms using filters designed based on experimentally obtained attenuation data. The waveforms are furthermore compared with AE signals obtained during quasi-static tensile testing of the same laminate along with dispersion curves to validate the FEM results.

## 2. Materials and Methods

### 2.1. Experimental Analysis

Several coupons of cross-ply carbon/epoxy laminate with lay-up sequence [0/90]_3s_ were loaded under quasi-static tensile load until failure. Acoustic emissions were monitored throughout the loading process using bonded PZT sensors to analyze the failure mechanisms. The detailed experimental procedure can be outlined as follows:

#### 2.1.1. Sample Preparation

Tensile test specimens were prepared according to ASTM Standard D3039 from carbon fiber-reinforced epoxy [0/90]_3s_ cross-ply laminates. The nominal dimensions for the tensile coupons were 12” × 1” × 0.071”. Glass–epoxy tabs were bonded to the ends of each specimen to facilitate gripping, with the tabs tapered at a 10° angle to minimize stress concentration at the interface.

#### 2.1.2. Instrumentation

**Figure 1:**
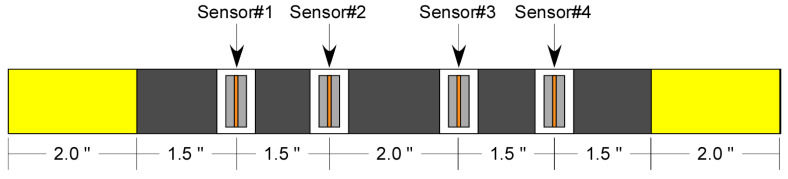
Schematic showing sensor position for carbon/epoxy cross-ply tensile coupons.

Bhuiyan et al. [27] have shown that while commercial AE sensors measure the low-frequency flexural modes and show very weak response to the axial (symmetric) modes, a piezoelectric wafer active sensor (PWAS) captures both axial and flexural modes of Lamb waves, hence making them suitable for AE monitoring of thin plate-like structures. Therefore, the acoustic emissions during the tensile test were recorded using surface-bonded PZT sensors with a sensing aperture of 1 mm at various locations, as seen in Figure 1. As discussed earlier, these surface-bonded PZT sensors are primarily strain-based sensors that are seen to provide high fidelity for Lamb waves.

#### 2.1.3. Loading

The sample was mounted in an MTS 810 material testing system and loaded monotonically with a loading rate of 300 lbs./min until failure.

#### 2.1.4. Data Acquisition

The acoustic emission waveforms were recorded using the PCI-2 data acquisition system, with signals amplified using PAC preamplifiers. A preamp gain of 60 dB and a sampling frequency of 20 MHz were used to record the AE waveforms. Additionally, a threshold of 40 dB and a bandpass analog filter with frequencies ranging from 1 kHz to 3 MHz were used to eliminate noise from the data. Several thousand waveforms were recorded during the experiment, with three characteristic waveforms appearing at various load levels.

**Figure 2:**
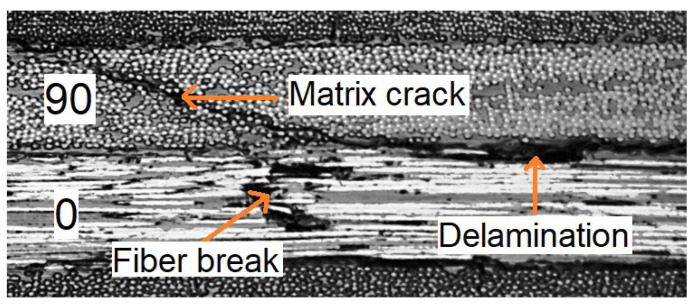
Micrograph of a cross-section of composite laminate showing different failure modes.

Figure 2 shows the micrograph of the cross-section of one of the failed specimens showing three failure modes. Matrix crack present in 90^0^ ply can be seen to grow as delamination. Similarly, fiber breaks can be seen in the 0^0^ ply.

**Figure 3:**
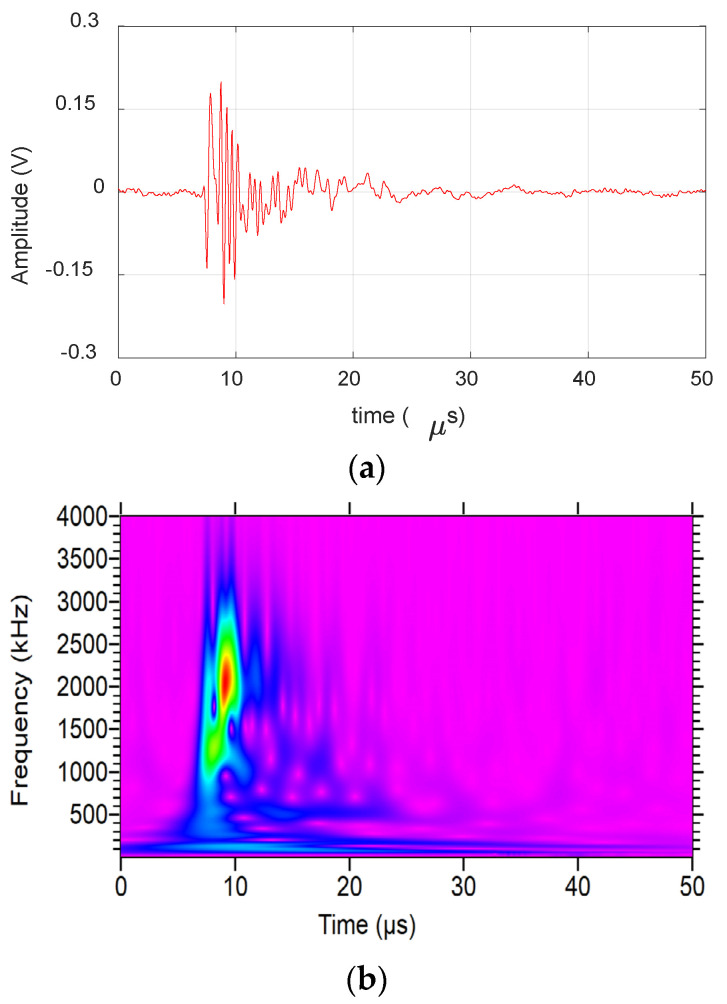
Fiber break event obtained during the experiment: (**a**) Waveform. (**b**) Wavelet transform.

Figure 3 shows an AE signal from a fiber break event obtained during the experimental analysis. The waveform can be characterized by short duration and amplitude with frequency content reaching up to 3 MHz. This presence of high-frequency content indicates the presence of higher-order Lamb wave modes, which are further analyzed in the numerical study.

**Figure 4:**
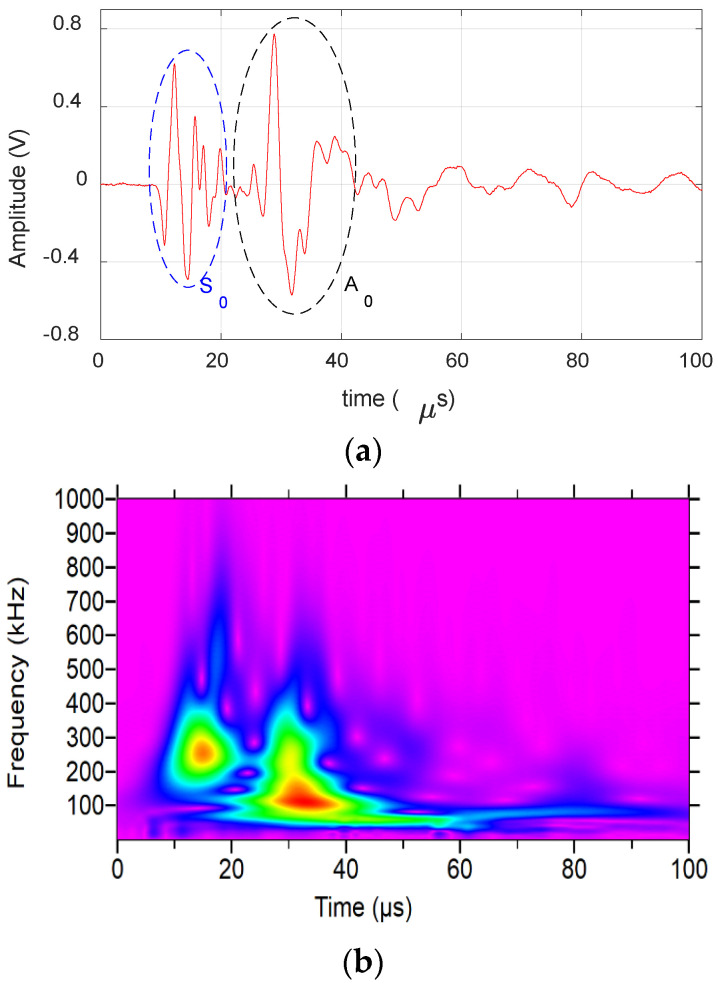
Matrix crack event obtained during the experiment: (**a**) Waveform. (**b**) Wavelet transform.

**Figure 5:**
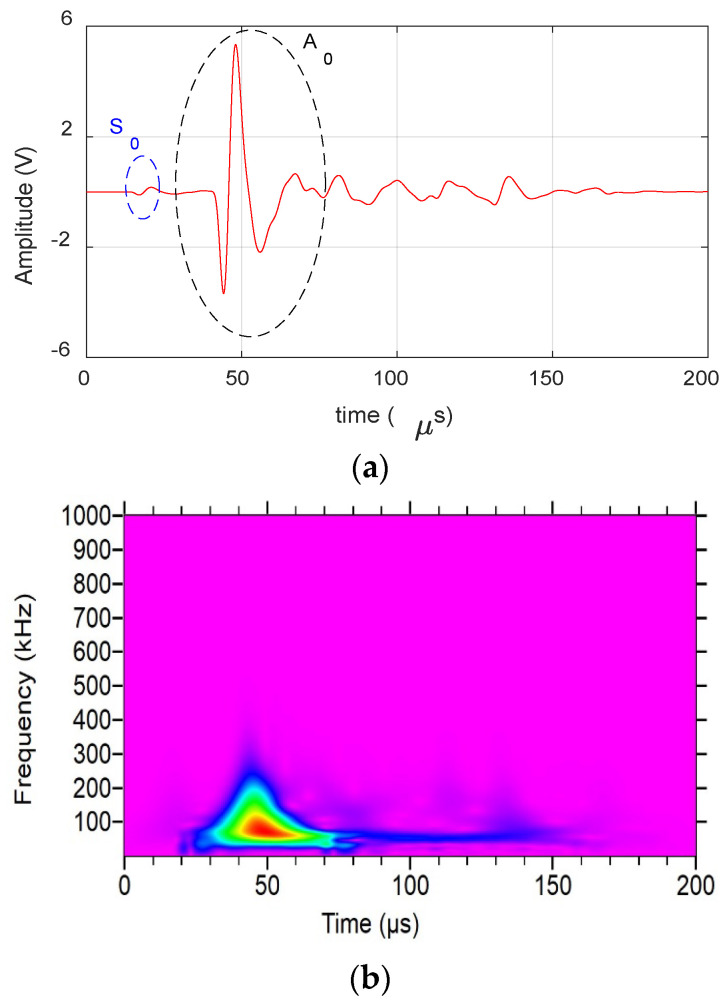
Delamination event obtained during the experiment: (**a**) Waveform. (**b**) Wavelet transform.

Figure 4 shows the AE waveform received from matrix crack events and its wavelet. The presence of S_0_ and A_0_ lamb wave modes can be clearly seen from the waveform and the wavelet. The S_0_ mode exhibits a low frequency arriving first, followed by a high frequency, whereas the A_0_ mode shows a high frequency arriving first, followed by a low frequency. This behavior is due to the differing group velocities of frequencies, as illustrated in the dispersion curve in Section 3. The waveform is also observed to have moderate amplitude and duration (<60 µs) with frequency extending up to 650 kHz. Similarly, Figure 5 shows the AE waveform received from a delamination event along with its wavelet. The dominant presence of A_0_ can be seen in both waveform and wavelet, with S_0_ mode buried in noise. The signal also has a high amplitude and very low-frequency content of <250 kHz, as seen in the figure.

### 2.2. Numerical Analysis

Finite element analysis was used to model wave propagation in a 2D composite plate model. Finite element analysis solves the dynamic equation of motion, as seen in Equation (1), by discretizing the domain of interest into finer elements, where [M] is the mass matrix, [K] is the stiffness matrix, and {F} is the load vector.

(1) $$\left[M\right]\left\{\ddot{u}\right\}+\left[K\right]\left\{u\right\}=\left\{F\right\}$$

For wave propagation analysis, FEM computes the elastodynamic equation of motion given by Equation (2), where µ and λ are Lame’s constant, u is the displacement at nodes, and f is the load vector.

(2) $$\mu u_{i,jj}+\left(\lambda +\mu \right)u_{j,ji}+\rho f_{i}=\rho \ddot{u_{i}}$$

A 2D cross-ply composite laminate with lay-up sequence [0/90]_3s_ and thickness of 1.8 mm was modeled using plane-strain 2D quad shell elements. Each laminate ply was modeled using orthotropic material properties, as seen in Table 1. These properties for 0^0^ laminate were also transformed using appropriate direction cosines and transformation matrices to match the respective ply orientation of 90^0^ and the material coordinate system of the FEM tool used (LS-DYNA).

**Figure 6:**
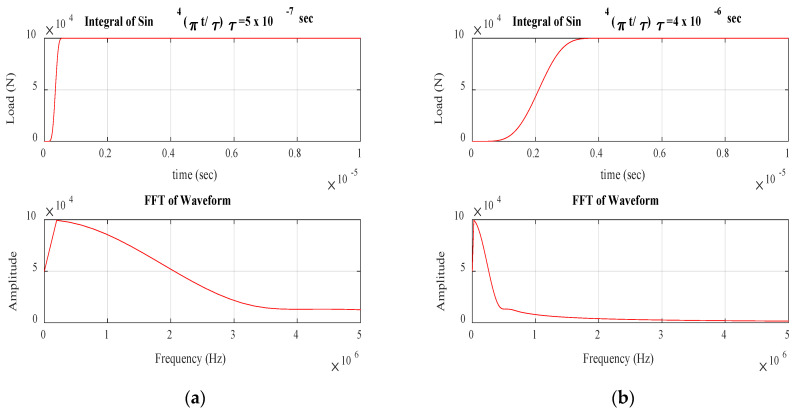

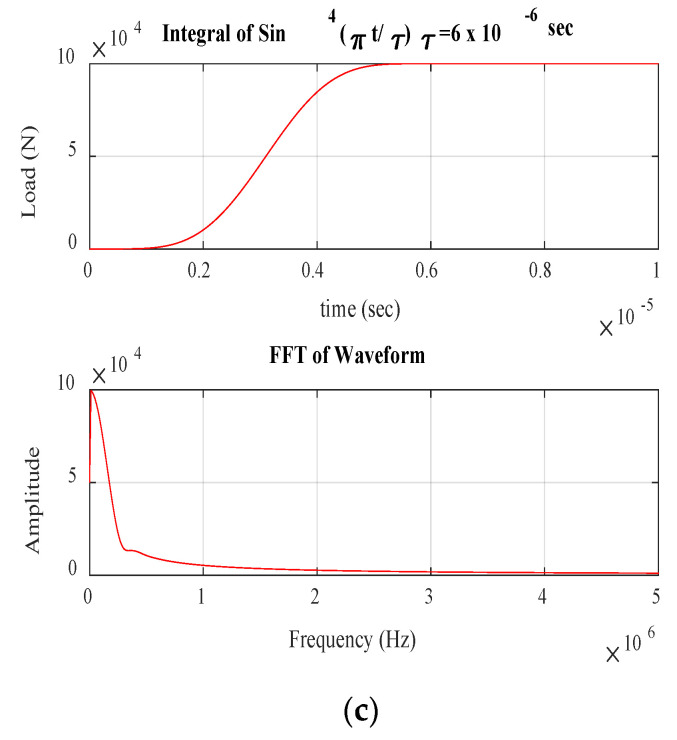
Source time functions and their FFTs used for simulating various failure modes: (**a**) Fiber break. (**b**) Matrix crack. (**c**) Delamination.

Figure 6 shows the various source time functions used as impulse loading to simulate failure modes under consideration. The duration of these source time functions is approximated based on the duration of AE source events considered. Fiber break events, having a short duration, were modeled with an integral of sine to power four with a very short rise time of 0.5 µsec, exceeding the frequency content of 3 MHz. Similarly, matrix crack and delamination were simulated using a longer-duration source time function with frequencies extending to 650 kHz and 250 kHz, respectively.

**Figure 7:**
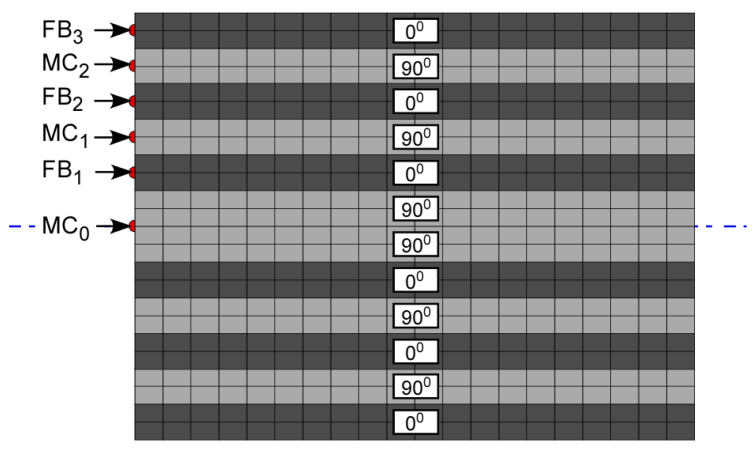
Schematics for various impulse loading showing locations of fiber break and matrix crack events.

**Figure 8:**
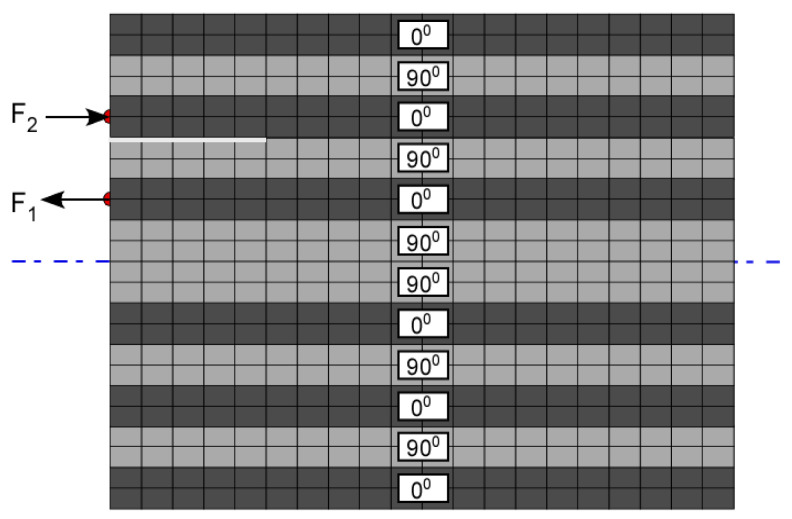
Schematics of couple loading for simulation of delamination events.

Figure 7 shows the schematic of various locations of impulses applied to model fiber break and matrix crack events. Different locations were used in individual models to study the variation in modal components of the waveforms received for the failure modes considered. With most delaminations developed from matrix cracks under the influence of in-plane shear force at the matrix crack tip, delamination was modeled as a Mode II fracture dominated by A_0_ Lamb waves [12]. Hence, a couple system, as seen in Figure 8, was used to simulate delamination in FEM models.

#### 2.2.1. Mesh Convergence

Mesh convergence analysis ensures that results from FEA are not affected by changing mesh size and, hence, is essential to establish mesh independence. H-refinement, where the element size is reduced, and p-refinement, where the element order is increased, are the two methods generally used for mesh convergence analysis [28]. In this study, h-refinement analysis is performed on a 2D composite bar under impulse loading, as detailed in Section 3, to ensure accurate propagation of applied impulse energy. The number of elements in each ply in the thickness direction was successively increased until the coefficient of variation in displacement and stresses was below 5%.

**Figure 9:**
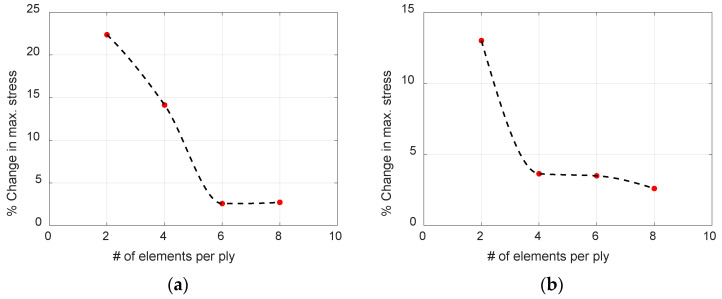
Percentage change in maximum axial stress in consecutive models with increasing number of elements in each lamina for (**a**) symmetric mode (**b**) anti-symmetric mode.

Figure 9 shows the percentage change in maximum axial stress when the number of elements per lamina gradually increases for both symmetric and anti-symmetric modes for the fiber break model, which requires the most spatial resolution due to the high-frequency content. To ensure optimal run time and number of elements, each model is meshed using six elements per lamina as the ideal percentage change of less than 5% is observed for models with six elements per ply for both symmetric and anti-symmetric mode.

#### 2.2.2. Attenuation in Thermoset Composites

**Figure 10:**
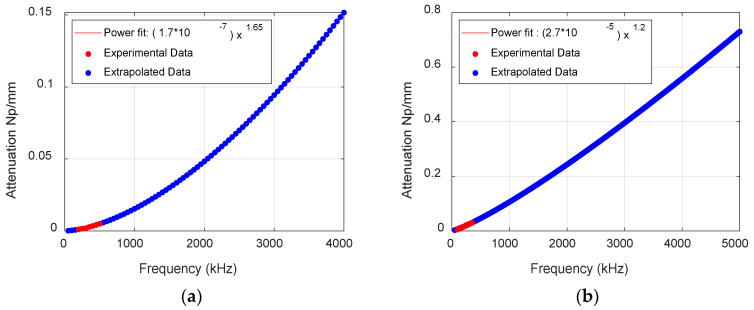
Experimental and extrapolated attenuation values along 0^0^ for (**a**) symmetric and (**b**) anti-symmetric modes.

**Figure 11:**
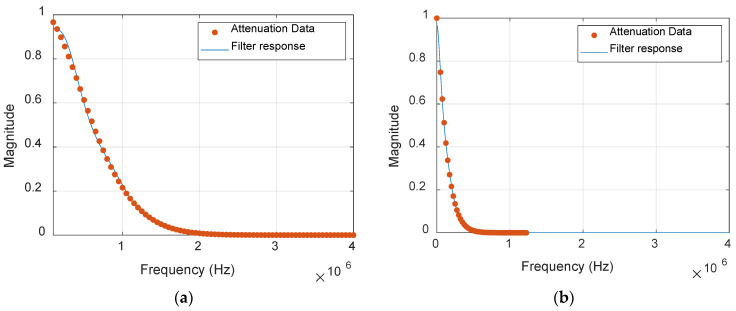
Attenuation values and the response of arbitrary amplitude filters along 0^0^ at 100 mm for (**a**) symmetric and (**b**) anti-symmetric modes.

Frequency-dependent attenuation was incorporated in the post-processing of FEM waveforms by designing and applying an arbitrary magnitude response filter to match the attenuation values, as seen in Figure 10. The attenuation measurement was taken by exciting the fundamental Lamb wave modes in the cross-ply laminate of layup [0/90]_3s_ using burst signals of frequencies ranging from 100 kHz to 500 kHz and measuring the signals at various distances and angles [15]. The attenuation values measured from the experiments for these frequencies were then extrapolated to obtain the values for higher frequencies. While the higher-order modes are challenging to excite and highly attenuated compared to fundamental modes, the same attenuation was applied for all symmetric and anti-symmetric modes. The attenuation values and response of the filter for the waveform received at 100 mm from the source are seen in Figure 11.

## 3. Results and Discussion

**Figure 12:**
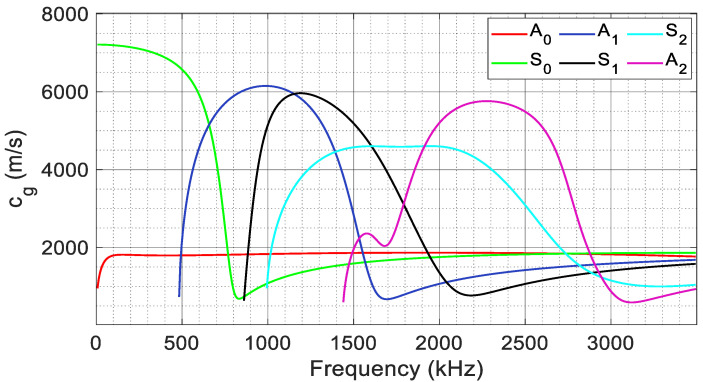
Dispersion curve showing group velocity for [0/90]_3s_ laminate used obtained from post-processing the results from GUIGUW software (ver-2.2) [29].

Figure 12 displays the dispersion curves for the group velocities of various Lamb wave modes in the thermoset composite examined in this study. These curves illustrate the wave propagation velocities of different mode shapes within the materials and serve as a tool to validate the results obtained from the finite element analysis.

**Figure 13:**
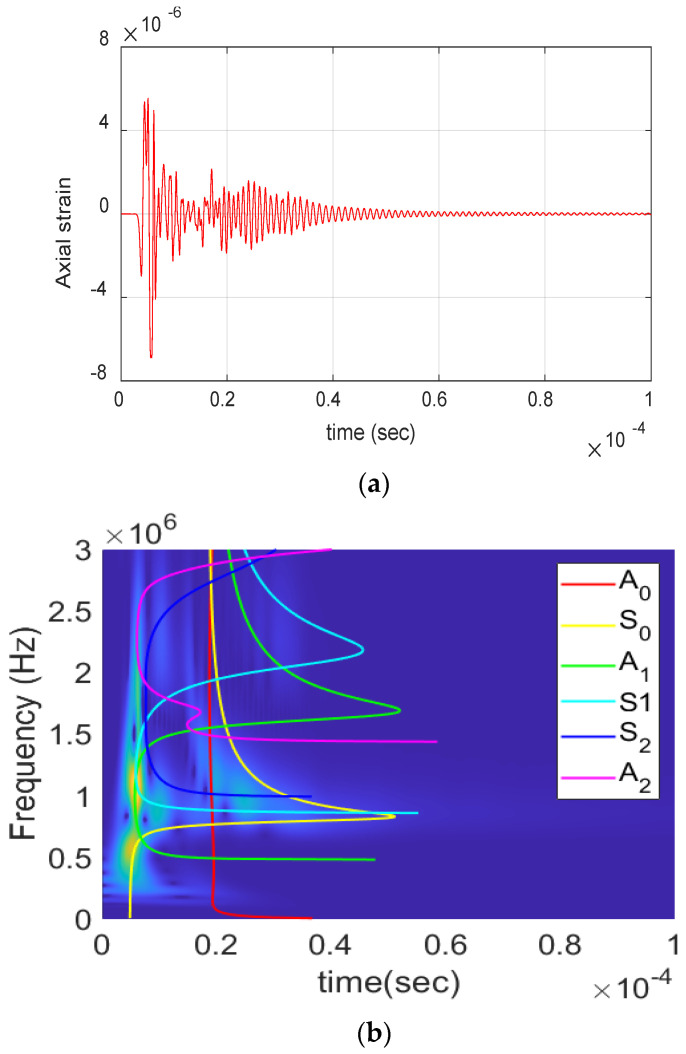
Simulated FB event obtained at 25 mm from the source at impulse location FB_2_: (**a**) Axial strain. (**b**) Wavelet fitted with dispersion curve.

Waveforms collected at different source-to-sensor distances were derived from several models with varying impulse locations, as discussed in Section 2.2. These waveforms reflect different types of failures at various locations. Filters were applied to introduce mode and frequency-based attenuation, reducing the amplitude of these waveforms. Figure 13 shows the attenuated waveform received at 25 mm from fiber break models with impulse applied at location FB_2_. The wavelet diagram fitted with the dispersion curve also shows the significant presence of higher-order modes in fiber break events with frequency content exceeding 2 MHz. Additionally, as seen from the wavelet, only the frequencies of individual modes where the slope of the dispersion curve is zero are observed in the waveforms. These frequency components possess similar velocities, causing them to superimpose and produce significant amplitude at these frequencies.

**Figure 14:**
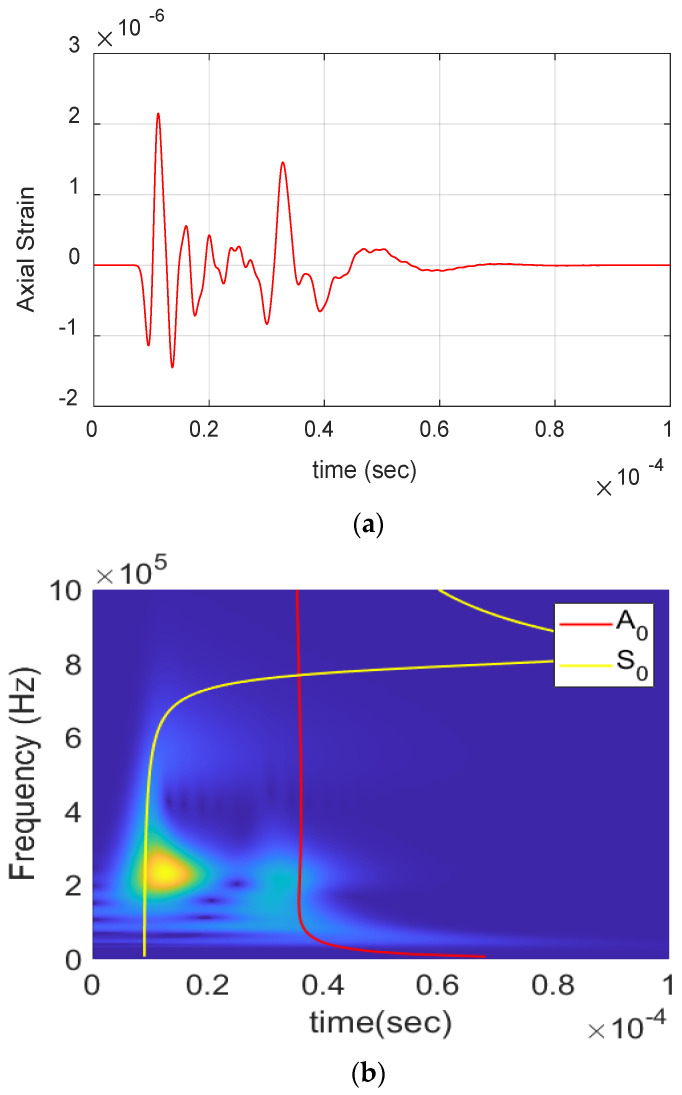
Simulated MC event obtained at 50 mm from the source at impulse location MC_2_: (**a**) Axial strain. (**b**) Wavelet fitted with dispersion curve.

**Figure 15:**
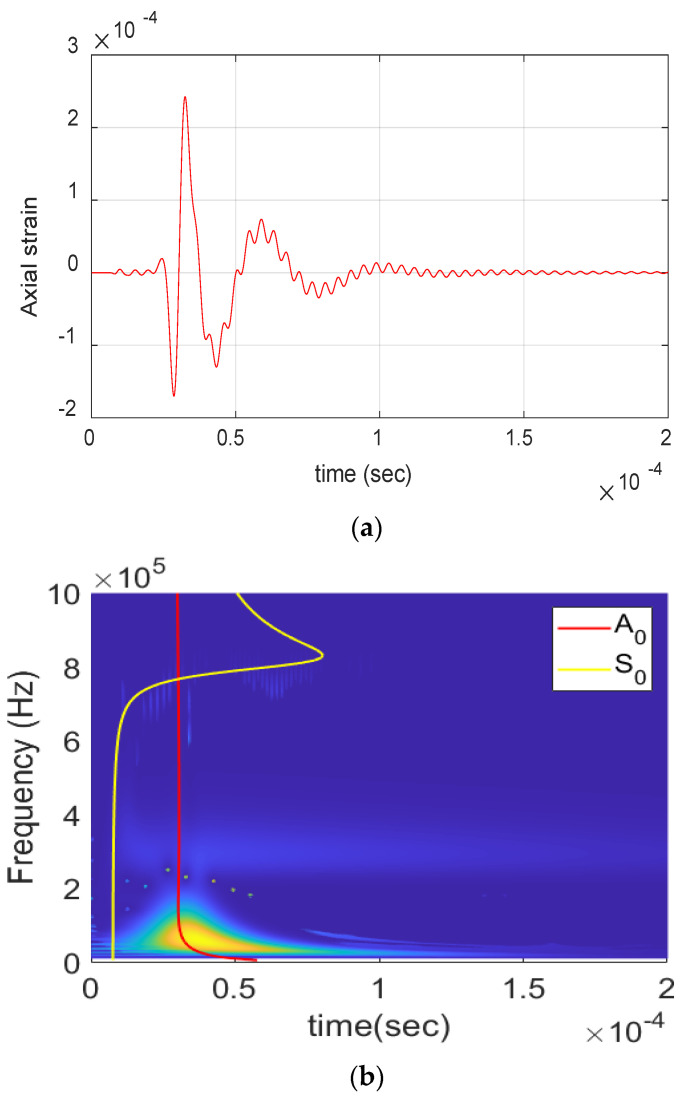
Simulated delamination event obtained at 50 mm from the source: (**a**) Axial strain. (**b**) Wavelet fitted with dispersion curve.

Figure 14 shows the attenuated waveform received at 50 mm for a matrix crack event located at MC_2_, along with the wavelet fitted with the dispersion curve. The presence of fundamental symmetric and anti-symmetric modes is clear from both the waveform and the wavelet. Likewise, the accurate orientation of dispersion curves in the wavelet supports the validity of the FEM models. Similarly, Figure 15 shows the attenuated waveform received at 50 mm from the impulse location for the delamination model. The wavelet shows a dominant A_0_ with a minimal S_0_, as seen in the leading edge of the waveform.

**Figure 16:**
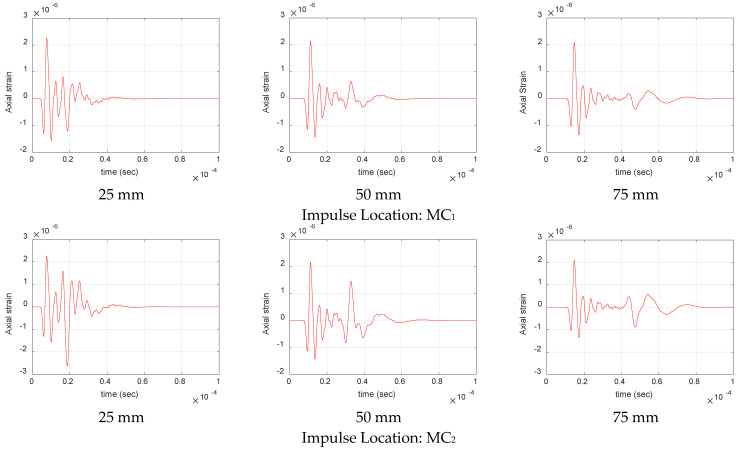
Waveforms for matrix crack at increasing source to sensor distance for different impulse locations in thermoset cross-ply composite.

Figure 16 shows the respective change in amplitude of S_0_ and A_0_ mode of matrix crack with distance for source located at MC_1_ and MC_2_. As seen from the figure, the amplitude of S_0_ mode, given by the leading edge of the signal, is similar for both offset positions. In contrast, a significant difference in the amplitude of A_0_ mode, given by the trailing edge of the signal, can be seen. The amplitude of the A_0_ mode is seen to increase as the distance of the source from the neutral axis increases, as seen from the waveform at 25 mm for MC_1_ and MC_2_. This rise in amplitude is primarily attributed to the increased bending moment, which generates anti-symmetric modes as the distance of the source from the neutral axis increases. Similarly, a substantial drop in amplitude of A_0_ mode compared to S_0_ can also be seen as anti-symmetric modes are highly attenuated compared to symmetric modes, as seen from the attenuation plots in Figure 10. This significant difference in attenuation is mainly due to the nature of excitation of symmetric and anti-symmetric modes. The anti-symmetric modes generate greater out-of-plane displacements at the material’s surface, increasing the possibility of energy leakage into the surrounding media. Similarly, these modes typically have higher strain energy because of their out-of-plane motion, leading to more attenuation, as energy dissipation is proportional to the strain amplitude. In contrast, the symmetric modes have in-plane motion, where particle displacements are largely along the direction of propagation and have less surface interaction. Additionally, the in-plane motion produces a more uniform displacement field, leading to lower strain gradients compared to anti-symmetric modes and hence resulting in lower attenuation [30].

**Figure 17:**
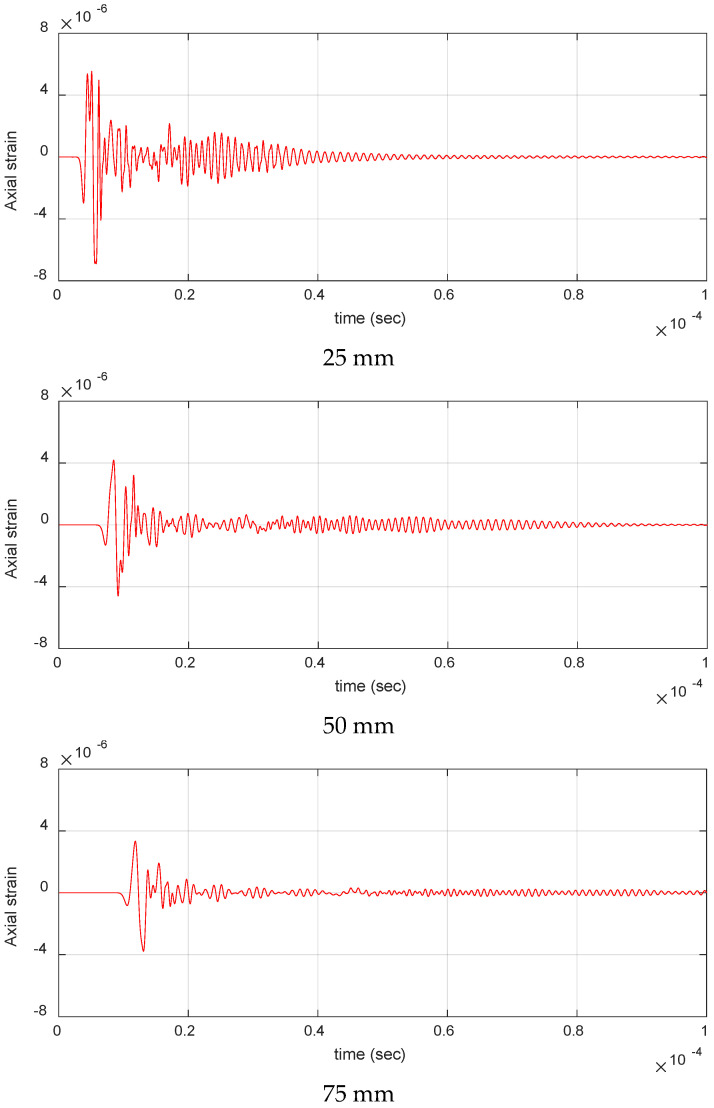
Waveforms for fiber break at increasing source to sensor distance for impulse located at FB_2_ in thermoset cross-ply composite.

Similarly, Figure 17 shows the change in waveforms with distance for fiber break events for impulse located at FB_2_. The high-frequency content of the signal at 25 mm indicates the presence of a higher-order mode in fiber break signals. As the source-to-sensor distance increases, amplitude and frequency content in the signals decrease, making the position of AE sensors crucial to capture these signals appropriately. As the damage mechanisms in pristine specimens are randomly distributed along the entire gage length, a dense array of AE sensors, as seen in Figure 1, is essential in capturing these events effectively.

### 3.1. Variation in AE Waveform Energy

**Figure 18:**
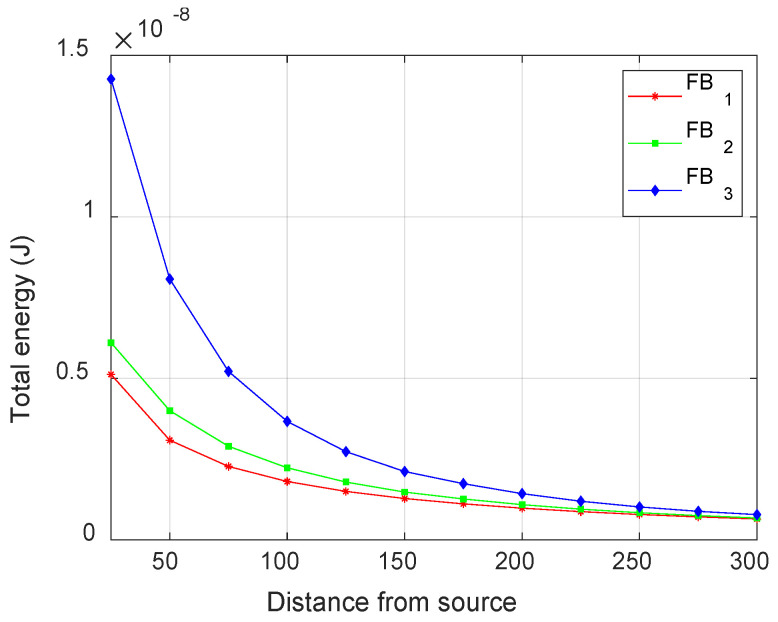
Variation of total attenuated energy with distance from the source for different impulse locations for fiber break.

The energy content of the waveform, represented by the area under the rectified and squared signal, was also calculated to study the variation in energy with increasing source-to-sensor distance for fiber break and matrix crack failure modes. While delamination events mainly give low-frequency signals (<200 kHz) that are less attenuated, the presence of mid-frequency (250–650 kHz) and high-frequency (>700 kHz) components in matrix crack and fiber break, respectively, make them highly susceptible to attenuation. Figure 18 shows the variation in signal energy for increasing source-to-sensor distance for the fiber break models considered. An exponential decrease in energy is evident across all three models, with the severity increasing as the events move further away from the neutral axis. This variation in energy is primarily attributed to the presence of anti-symmetric modes, which exhibit an increase in amplitude with an increase in distance from the neutral axis and are significantly more attenuated than symmetric modes.

**Figure 19:**
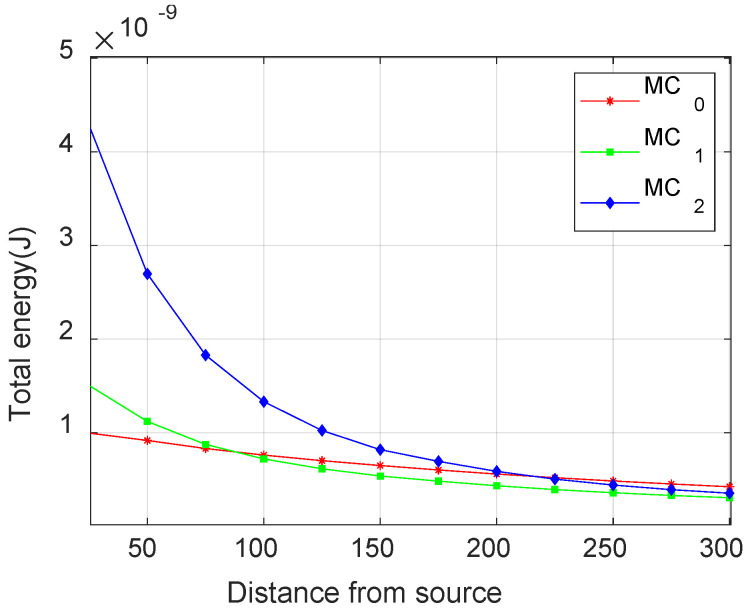
Variation of total attenuated energy with distance from the source for different impulse locations for matrix crack.

Similarly, Figure 19 shows the variation in energy of matrix crack events for increasing source-to-sensor distance at different locations. The matrix crack at the neutral axis (MC_0_) shows only symmetric modes, resulting in minimal energy variation due to the low attenuation of these modes. In contrast, a significant drop in energy is observed for the matrix crack event furthest from the neutral axis (MC_2_). This decrease is primarily due to the higher attenuation of anti-symmetric modes, as previously discussed.

### 3.2. Comparison of FEM and Experimental Waveforms

**Figure 20:**
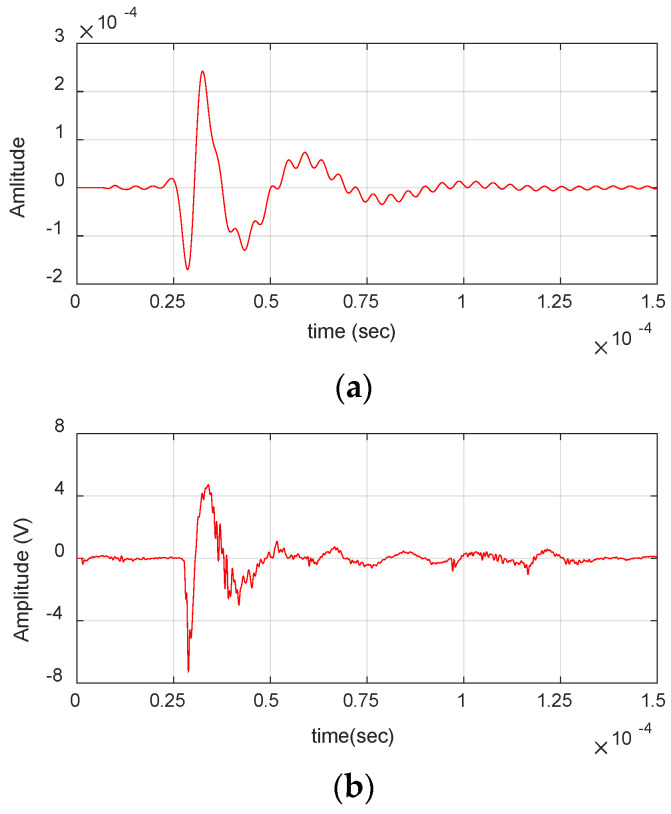
Comparison of waveforms from FEM and experiments for delamination: (**a**) FEM. (**b**) Experimental waveform (C.C 86%).

**Figure 21:**
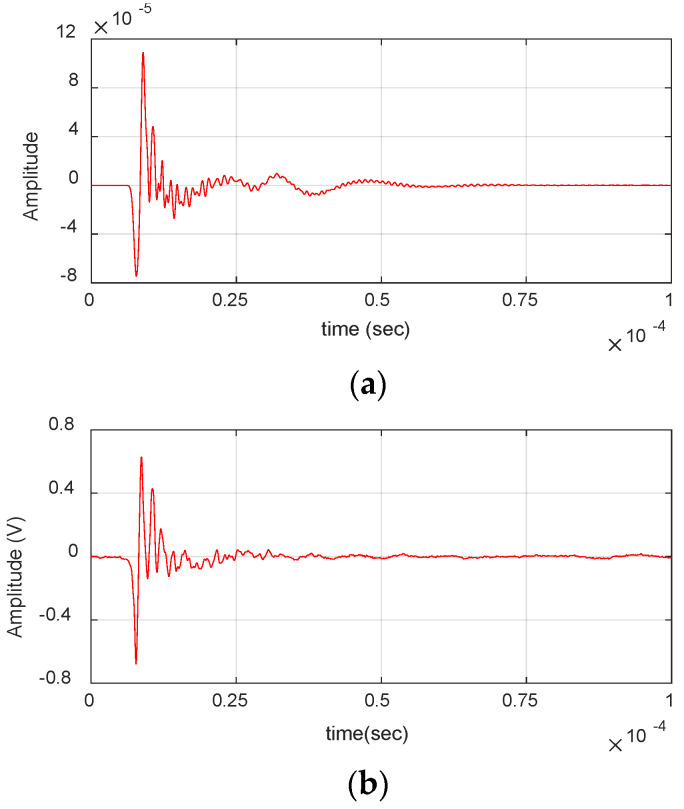
Comparison of waveforms from FEM and experiments for fiber break: (**a**) FEM. (**b**) Experimental waveform (C.C. 85%).

**Figure 22:**
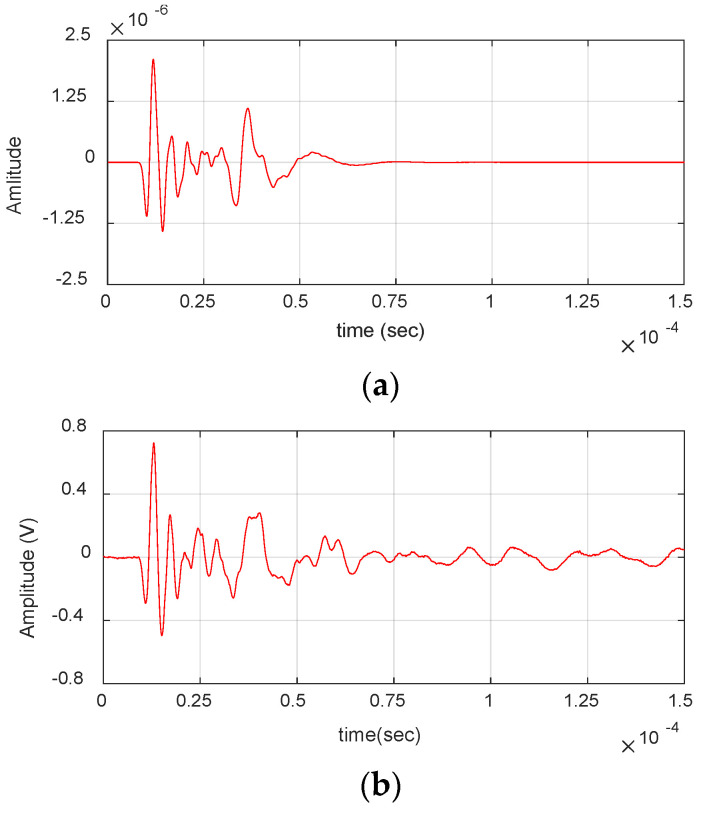
Comparison of waveforms from FEM and experiments for matrix crack: (**a**) FEM. (**b**) Experimental waveform (C.C 84%).

The FEM-simulated waveforms were further correlated with experimental AE waveforms obtained during the quasi-static tensile test of the same composite coupons. Figure 20 shows the simulated waveform for delamination and that from the experiments having a good correlation coefficient (C.C) of 86%. Similarly, Figure 21 and Figure 22 show the simulated waveform of fiber break and matrix crack along with the waveforms received from the experiments. A good correlation of 85% and 84% was observed in the simulated waveforms, respectively, indicating FEM can be used effectively to simulate the failure modes considered in this study. Although a reasonable correlation is observed between experimental and FEM-generated waveforms, the discrepancy can primarily be attributed to intrinsic electronic noise present in the experimental waveforms and the shape of the source time function used in FEM simulations.

## 4. Conclusions

In this study, finite element analysis was used to model the three primary failure modes seen in cross-ply carbon/epoxy laminates subjected to quasi-static tension. Failures occurring at different locations along the thickness of laminate and several source-to-sensor distances were considered. AE waveforms were modified by incorporating frequency and mode-dependent attenuation results from prior experimental work by post-processing and applying appropriate amplitude response filters to the FE-generated waveforms. These waveforms were further validated with dispersion curves. The results indicated that the fundamental symmetric (S_0_) and anti-symmetric (A_0_) modes predominantly governed the signals. Fiber break events displayed the fundamental modes along with higher-order Lamb wave modes exceeding 2 MHz in frequency. Depending on the crack location, matrix cracks were characterized by S_0_ modes or a combination of S_0_ and A_0_ modes and exhibited moderate frequencies. Delaminations were primarily represented by low-frequency A_0_ mode. Variations in velocities and attenuation among the different Lamb wave components caused notable changes in the waveforms at different source-to-sensor distances. Anti-symmetric modes, which are more attenuative than symmetric modes, significantly influenced the nature and energy of the signals recorded at different source-to-sensor distances. The substantial decrease in amplitude and frequency content associated with high-frequency failure modes like fiber break highlights the need for optimal sensor placement to capture these waveforms effectively, requiring a dense array of sensors to be used, as seen in the experiments. Furthermore, a reasonable correlation between the simulated waveforms and experimental acoustic emission signals indicates the effectiveness of FEA in modeling these failure modes in composite materials and complex geometries, where experimental methods may not be feasible.

## Figures and Tables

**Table 1:** Orthotropic material properties for 0-degree laminate.

E_11_ (Pa)	E_22_ = E_33_ (Pa)	G_12_ = G_13_ (Pa)	G_23_ (Pa)	$\nu_{12}=\nu_{13}$	$\nu_{23}$	$\rho \left(\mathrm{kg}/m^{3}\right)$
1.47 × 10^11^	10.3 × 10^9^	7 × 10^9^	3.7 × 10^9^	0.27	0.54	1520

## Data Availability

The original contributions presented in this study are included in the article. Further inquiries can be directed to the corresponding author.
